# Amino-induced cadmium metal–organic framework based on thiazole ligand as a heterogeneous catalyst for the epoxidation of alkenes

**DOI:** 10.1038/s41598-023-42666-1

**Published:** 2023-09-16

**Authors:** Fatemeh Moghadaskhou, Akram Karbalaee Hosseini, Azadeh Tadjarodi, Mehdi Abroudi

**Affiliations:** Research Laboratory of Inorganic Materials Synthesis, Department of Chemistry, Iran University of Science and Technology (IUST), Tehran, 16846-13114 Iran

**Keywords:** Chemistry, Catalysis

## Abstract

Selective epoxidation of olefins is of high interest in the chemical industry due to the many applications of epoxides. This study reports on the synthesis of Cd-MOF, [Cd(DPTTZ)(5-AIP)] (IUST-1) (where DPTTZ = 2, 5-di (pyridine-4-yl) thiazolo [5, 4-d] thiazole, 5-AIP = 5-Aminoisophthalic acid), by a reflux method, which can be considered as a fast and simple process. The morphology and structure of the synthesized IUST-1 were determined by using FE-SEM (Field Emission Scanning Electron Microscopy), EDX (Energy Dispersive Analysis of X-ray), Mapping (Elemental Mapping), CHNS (Elemental analysis), XRD (X-Ray Diffraction), FT-IR (Fourier Transform Infrared), and TGA (Thermo Gravimetric Analysis). The epoxidation of cyclooctene was investigated using the activity of catalytic IUST-1. The results showed that in the presence of tert-butyl hydroperoxide and CCl_4_ in a 1:2 alkene/oxidant ratio, a high epoxide yield (99.8%) was obtained. In addition, IUST-1 can be easily separated by simple filtration and recycled five times successfully with a slight decrease in activity. This compound has some advantages such as high yield, short reaction time, and ease of reuse, which make it a suitable heterogeneous catalyst for the epoxidation of cyclooctene.

Oxygen-atom transfer reactions have been studied in detail for decades to make a wide range of chemicals, including epoxides. Epoxides are three-membered cyclic ethers that have a highly strained ring and are very reactive^1^. Chemical intermediates and species such as epoxides play an important role in the production of pharmaceuticals, agrochemicals, and relevant industrial chemicals^2^. Many studies have been conducted on homogeneous and heterogeneous catalysts to catalyze the oxidation of olefins to fine chemicals due to their industrial relevance. The benefits of the use of catalysts in chemical processes are increasing the efficiency of products, reducing the by-products, and reducing the required temperature, as well as increasing the reaction selectivity^3–6^. Oxidation of alkenes to the corresponding epoxides is conducted through the chlorohydrin process or is investigated using m-chloroperoxybenzoic acid (mCPBA), tert-butyl hydroperoxide (TBHP), and hydrogen peroxide (H_2_O_2_)^7^. There is a challenge in obtaining high selectivity and enantioselectivity in epoxidation reactions.

A new class of crystalline solids is formed through the cooperative arrangement of metal ions or clusters (called secondary building units) with organic ligands called porous coordination polymers (PCPs) or metal–organic frameworks (MOFs). Diverse compositions and topological structures make MOFs highly suitable for numerous applications, including catalysis^8,9^, adsorption^10,11^, magnetism^12,13^, electrode material^14^, luminescence^15,16^, biomedical^17^, gas storage^18^, and separation^19^. There has been a great deal of research and development on the use of MOFs as catalysts, including the development of fine chemicals^20^, and the developing of green protocols that could substitute non-eco-friendly catalysts^21^. It has been demonstrated that MOF structure significantly impacts activity and selectivity for specific organic reactions^22^. MOFs have been designed using organic linkers and catalytically active inorganic ions and exerted as heterogeneous catalysts with no requirement of immobilization on solid supports or post-synthetic modifications^23–25^. The main advantage of MOFs in catalysis is their ability to be designed and predicted based on the linker features, geometry of the metal, and coordination number. MOFs used as heterogeneous catalysts have attracted significant attention in the oxidation of olefins. This is because of having high-potential active sites such as modified ligands with active sites, metal centers with exchangeable coordination positions, and empty cavities with active species^26^.

In spite of the numerous reported catalytic uses of MOFs, the development of truly efficient and selective catalytic processes using MOFs remains a challenge. Some MOFs have low stability and are susceptible to self-decompose in organic solvents and water, which limits their use as photocatalysis, biocatalysis, and electrocatalysis^27^. Moreover, the ability to obtain monodisperse nano‐sized MOFs is of major importance for prospective applications in heterogeneous catalysis^28^.

In this work, the reflux method was used to prepare an amino-functionalized Cd-MOF (IUST-1) based on the thiazole ligand in a fast, simple, and promising manner for a large-scale. IUST-1 displays an excellent ability in the epoxidation of olefins. The catalytic properties of IUST-1 for the epoxidation of cyclooctene were investigated using different reaction parameters, such as temperature, catalyst amount, oxidant, reaction time, and solvent effect. The IUST-1 demonstrated a high yield (99.8%) in the epoxidation of cyclooctene in CCl_4_ in the presence of TBHP in a 1:2 alkene/oxidant ratio. In addition, the IUST-1 catalyst can be used for the oxidation of various olefins with high efficiency.

## Experimental

### Materials and measurements

All chemicals and solvents used in the syntheses were of analytical reagent grade and were used without further purification. Elemental analysis (C, H, N, and S) was carried out on a Thermo Finniga Flash 1112 series elemental analyzer. FT-IR spectra were recorded using the KBr pellet method on a Shimaduz FT-IR-8400 spectrometer. Power X-ray diffraction (PXRD) was conducted by a Philips X’pert X-ray powder diffractometer (Cu-Kα, *λ* = 1.5418 Å). TGA analysis was carried out using Perkin Elmer Pyris 1 thermo gravimeter under an argon (Ar) atmosphere in the range from 50 to 800 °C with a ramp rate of 10 °C.min^-1^. Field emission scanning electron microscope (FE-SEM) measurement was performed using the FE-SEM TESCAN MIRA3 microscope. GC–MS samples were registered on a Shimadzu QP-5050 GC–MS device.

The leaching metals of catalyst was analyzed using ICP-MS (Inductively coupled plasma mass spectrometry) on a Vavian 715-ES.

### Preparation of nano-sized (IUST-1)

A solution of 2, 5-di (pyridine-4-yl) thiazolo [5, 4-d] thiazole (DPTTZ) (25 mg, 0.08 mmol), 5-Aminoisophthalic acid (5-AIP) (30 mg, 0.71 mmol), and Cd (NO_3_)_2_‧4H_2_O (50 mg, 0.17 mmol) in dimethyl formamide (DMF) (30mL) was added into a 50-mL round-bottomed flask equipped with a reflux condenser and a magnetic stirring bar. The reaction mixture was refluxed at 120 °C for 12 h. After cooling the reaction mixture, the resulting precipitate was filtered, washed several times with DMF, and then dried in the oven. FT-IR (KBr, cm^−1^): 3355 (m), 3258 (s), 3058 (w), 2925 (w), 1606(s), 1547 (s), 1417 (s), 1381 (s), 1321 (s) 1241 (s), 1066 (s), 1024 (s), 826 (s), 780 (s), 735 (s), 705 (s), 664 (s), 620 (s), 503 (s). Elemental Anal. calc. for C_22_H_13_CdN_5_O_4_S_2_: C, 44.94; H, 2.23; N, 11.91; S, 10.91%. Found: C, 45.65; H, 2.34; N, 11.63; S, 10.78%.

### Catalyst reaction

#### Typical catalytic method for selective aerobic oxidation of cyclooctene

Under the following conditions, the catalytic reaction was carried out: a heterogeneous Cd (II) catalyst (IUST-1) (20 mg), ammonium mono vanadium (NH_4_VO_3_) (4 mg) as cocatalyst, tert-butyl hydroperoxide (TBHP) 0.20 ml) as oxidant, carbon tetrachloride (0.50 ml) as solvent, and cyclooctene (0.13 mL). Two hours were required to complete the reaction at 76 °C. After centrifuging the solution, the filtered liquid samples were analyzed by gas chromatography-mass spectrometry (Shimadzu QP-5050 GC–MS system).

#### Catalyst recycling

Testing of catalyst recycling involved filtering, washing with ethanol, and drying at 80°C. It can then be reused for up to five runs under similar conditions.

#### Leaching test

After the catalytic reaction, ICP analysis was performed to measure the cadmium concentration in the solution and to determine whether cadmium metal might leach under the reaction conditions.

## Results and discussion

### Characterization of the IUST-1

**Figure 1:**
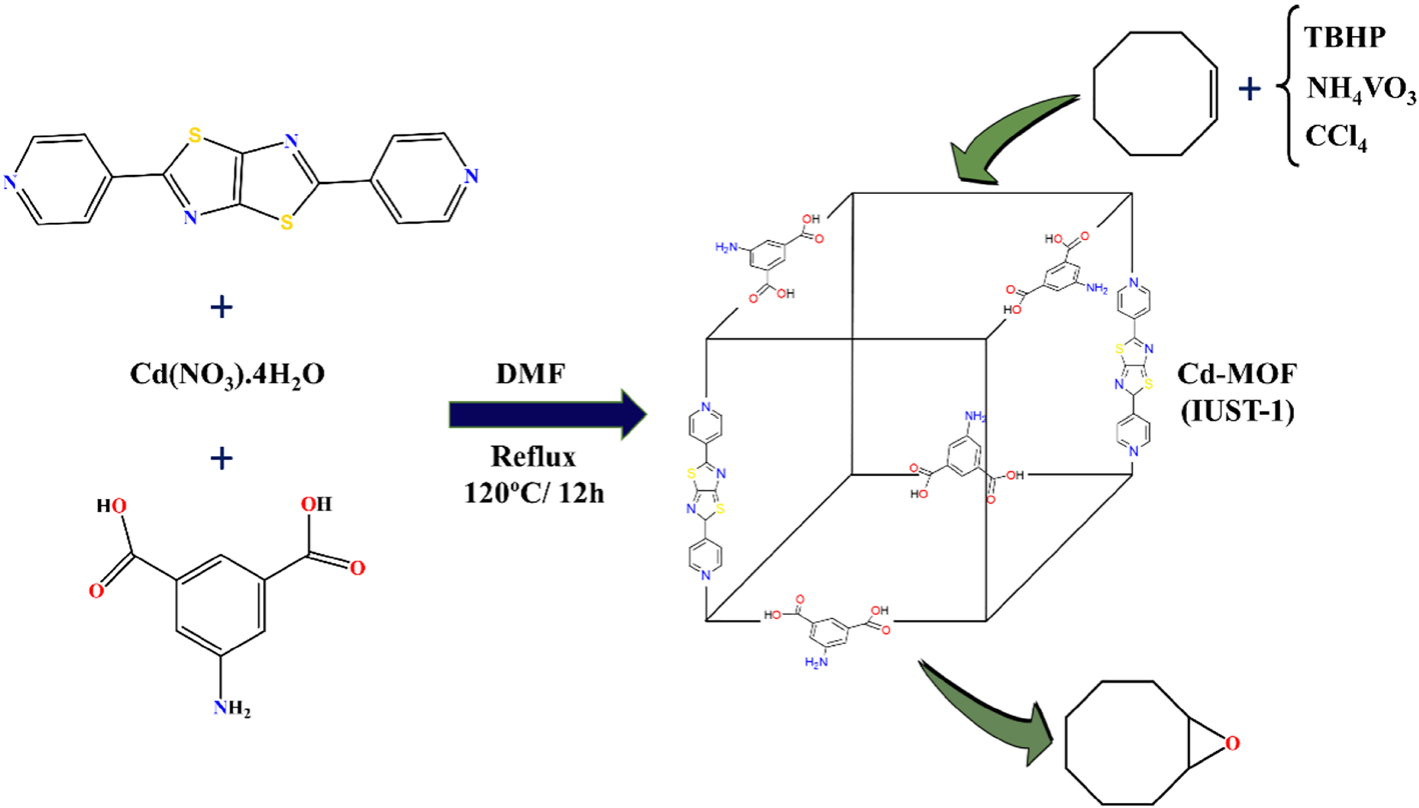
The schematic diagram for the preparation of the IUST-1 and the epoxidation of cyclooctene by it.

Using a reflux method, 5-amino isophthalic acid, 2, 5-di-pyridine-4-yl-thiazolo [5, 4-d] thiazole, and cadmium nitrate tetrahydrate were combined to produce IUST-1 (Fig. 1). The X-ray diffraction analysis exhibits that IUST-1 has a space group of Pbam and crystallizes in the orthorhombic crystal system. The central Cd(II) ion is seven-coordinated with five equatorial oxygen donors of carboxylate groups from three different 5-AIP ligands and two axial nitrogen donors of DPTTZ ligands, indicating a distorted [CdN_2_O_5_] pentagonal bipyramidal geometry. The IUST-1 is based on binuclear Cd(II) units, Cd_2_ (μ-OCO)_2_, that is 6-coordinated and linked through 5-AIP and DPTTZ ligands. By coordinating the 5-AIP and linear DPTTZ ligands, a 3D framework is created.

Figure S1 shows the simulated powder X-ray diffraction (PXRD) pattern from single crystal X-ray data of IUST-1 in comparison with the PXRD pattern of a nano-particles typical sample of IUST-1 prepared by the reflux process. The prepared IUST-1 by the reflux method exhibits a good agreement with the simulated model on its PXRD pattern, demonstrating its phase purity. On FT-IR spectra of IUST-1 (see supplementary Fig. S2 online), the vibrational bands for the primary amine (NH_2_) of 5-Aminoisophthalic acid as a ligand can be seen at 3355 and 3258 cm^−1^. The asymmetric and symmetric vibrations of the dicarboxylate groups of the 5-Aminoisophthalic acid ligand appear at 1606 and 1381 cm^−1^, respectively. In the reaction with Cd ions, all carboxyl groups of 5-Aminoisophthalic acid ligands have been deprotonated, as suggested by the absence of the expected absorption bands at 1700 cm^−1^. As part of the thermal gravimetric analysis (TGA) of the nanostructured IUST-1 synthesized by the reflux process, the thermal stability is tested between 50 and 800 °C underflows of argon (see supplementary Fig. S3 online). Thermal analysis results determined that the overall thermal stability of the IUST-1 prepared by the reflux process is similar to those presented in the literature^29^. The IUST-1 prepared by the reflux process shows stability up to approximately 370 °C. The adsorption–desorption isotherm of the IUST and BET results have been given in Fig. S4. According to this figure, the IUST is a mesoporous compound and the average size of pores is 32 nm.

**Figure 2:**
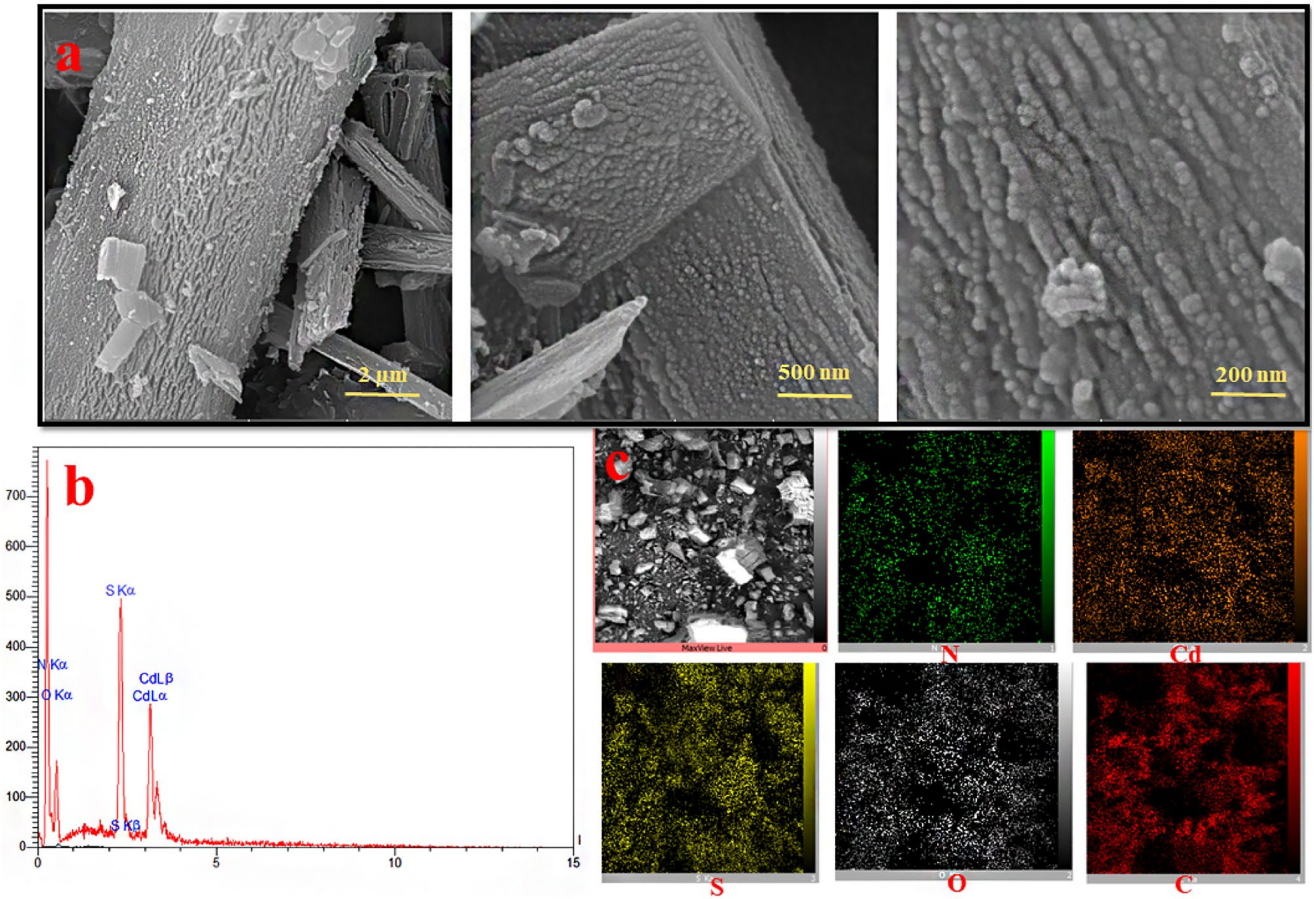
(**a**) FE-SEM images, (**b**) EDAX, and (**c**) Mapping analysis for the IUST-1 prepared by the reflux process.

The FE-SEM images of the reflux-produced IUST-1 have be given in Fig. 2. In the FE-SEM photographs, the structure of IUST-1 can be seen as a series of nanoplates formed by juxtaposing nanoparticles.

### Catalytic activity of the IUST-1 for epoxidation of cyclooctene

In this part of the study, the catalytic activity of IUST-1 in the epoxidation of cyclooctene was investigated. This reaction is one of the most commonly used procedures in biomedical and pharmaceutical synthesis. To begin with, the impact of temperature on the epoxidation of cyclooctene was investigated to identify the optimal conditions (Table 1, Entries 1–4).

**Table 1:** The effect of different temperatures and oxidants on the catalytic reaction^a^.

Entry	Temperature (°C)	Oxidant	Yield (%)
1	Room temperature	TBHP	0
2	60	TBHP	73
3	40	TBHP	35
4	**76**	**TBHP**	**99.8**
5	76	H_2_O_2_	10
6	76	NaIO_4_	0

^a^Reaction conditions: cyclooctene (1 mmol), 20mg IUST-1 as a catalyst, CCl_4_ (0.5 mL), NH_4_VO_3_ (4mg), oxidant (2 mmol), 2 h.

Significant values are given in Bold.

In this part, it is found that higher temperatures give higher yields. As the temperature increases, the kinetic energy increases, and the number of interactions between the initial materials increases. Thus, 76°C is optimal temperature for cyclooctene epoxidation in the presence of the IUST-1.

By comparing all three oxidants, it can be seen that TBHP obtains a higher product efficiency. The reason may be that there is peroxide oxygen in TBHP, which has a greater electrophilic character^30^.

Also, TBHP has a benefit compared to H_2_O_2_ due to the absence of water formation resulting from the reduction of this oxidant. Water is indeed considered to be at the origin of the IUST-1 catalyst deactivation (Table 1, Entries 4–6). In many cyclooctene oxidation reactions, Using TBHP as an oxidant, the reaction leads to a mixture of compounds but with the use of the IUST-1 catalyst, no side product was created and the creation of a single product in the catalytic reaction is a very important advantage^31,32^.

In the following step, the catalyst value and time were optimized (see supplementary Tables S1 and S2 online) and 20 mg of the IUST-1 and time 2 h were obtained as the optimal conditions of catalyst for the epoxidation of cyclooctene.

In the epoxidation of cyclooctene, solvents play a critical role in determining the rate and yield of products. In order to obtain the best solvent for epoxidation cyclooctene with the IUST-1 and NH_4_VO_3_ as co-catalysts, some reactions were performed (Table 2). Based on the results, carbon tetrachloride (Table 2, Entry 2) is the best solvent with a 99.8% yield. In comparison with carbon tetrachloride, other solvents like DMF, methanol, ethanol, acetonitrile, and acetone (Table 2, Entries 4–9) show lower yields and almost 0%. Furthermore, 1,2-dichloroethane (CH_2_Cl_2 )_ had relatively good yields in the product (Table 2, Entry 3), but carbon tetrachloride was selected as the solvent in the end. In the absence of the catalyst, no cyclooctene conversion was observed (Table 2, Entries 1).

**Table 2:** The impact of diverse solvents on the oxidation reaction cyclooctene^a^.

Entry	Catalyst	Solvent	Yield (%)
1	None Catalyst	1,2-Dichloroethane or Carbon Tetrachloride	0
2	IUST-1	Carbon Tetrachloride	99.8
3	IUST-1	1,2-Dichloroethane	98
4	IUST-1	Acetonitrile	8
5	IUST-1	Methanol	0
6	IUST-1	Ethanol	3
7	IUST-1	Acetone	0
8	IUST-1	Dimethyl formamide	0
9	IUST-1	Solvent-free	8

^a^Reaction conditions: cyclooctene (1 mmol), 20mg IUST-1 as a catalyst, solvent (0.5 mL), NH_4_VO_3_ (4mg), TBHP (2 mmol), 76 °C, 2 h.

The reported solvents in Table 2 due to their stability in oxidation processes were selected. Moreover, the results indicate that cyclooctene exhibits more reactivity in CH_2_Cl_2_ and CCl_4_ rather than CH_3_CN and other polar solvents. This can be explained by the coordination ability of CH_3_CN that inhibits the reaction by competing with the oxidant to occupy the coordination sites of the catalyst. These results are consistent with the previous reports^30^.

The IUST-1 was investigated as a catalyst in the epoxidation of alkenes using different olefins (Table 3). This catalyst displays a good catalytic performance for the epoxidation of various olefins.

**Table 3:** Substrate scope for epoxidation reaction^a^ of olefins using the IUST-1 as a catalyst.

Alkene	Substrate	Time(min)	Yield%	TON^b^	TOF^c^
Cyclooctene		120	99.8	29.35	14.67
Cyclohexene		120	93	27.35	13.68
Alpha-Methyl styrene		150	90	26.47	10.50
Styrene		180	85	25	8.33
1-Hexene		270	72	21.17	4.70
1-Octene		270	70	20.59	4.57
Alpha-Pinene		180	85	25	8.33
2-methylstyrene		300	88	25.88	5.18
4-Methylstyrene		180	90	26.47	8.82
trans-Stilbene		360	93	27.35	4.56
4-Chlorostyrene		330	91	26.76	4.86
4-Nitrostyrene		330	87	25.59	4.65
Cyclopentene		210	94	27.64	7.90

^a^Reaction conditions: Substrate (1 mmol), 20mg IUST-1 as a catalyst, CCl_4_ (0.5 mL), NH_4_VO_3_ (4mg), TBHP (2 mmol), 76 °C.

^b^TON = (mol of substrate /mol of catalyst)*yield of product^33^.

^c^TOF = TON/Time (h)^33^.

NH_4_VO_3_ was used as a co-catalysts in order to obtain the best yield for epoxidation cyclooctene with the IUST-1. After performing GC/Mass analysis, the results showed the necessity of NH_4_VO_3_ use (see supplementary Table S3 online). In the presence of only the IUST-1, the product yield was 53%, but with co-catalyst (NH_4_VO_3_), the product yield was 99.8%.

### Leaching and recycling tests

**Figure 3:**
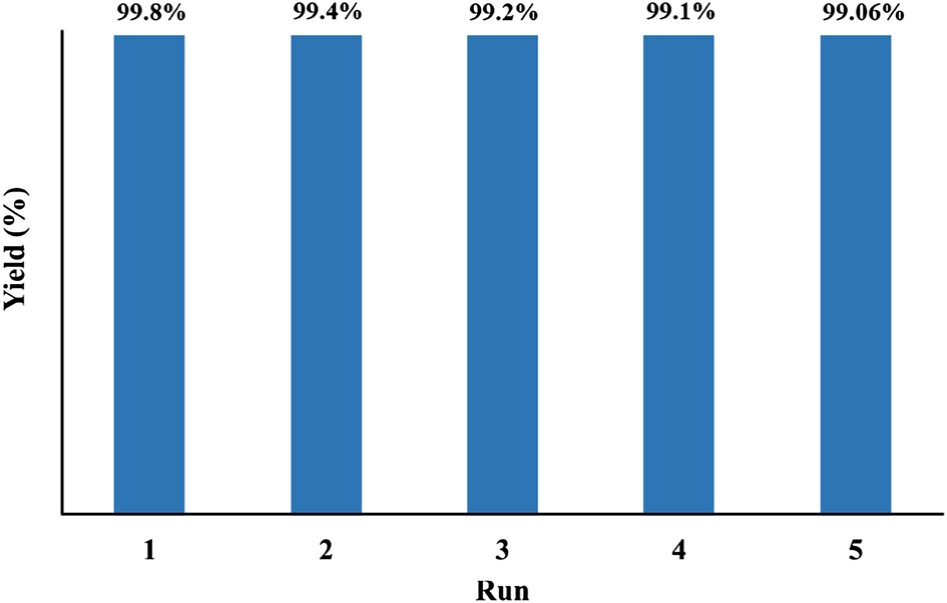
Reusability of the IUST-1catalyst in the epoxidation of cyclooctene.

To confirm the catalytic nature of IUST-1, the leaching and recycling tests were performed. The Cadmium-based catalyst was tested for its reusability for cyclooctene epoxidations. At the end of the reaction, the catalyst was centrifuged, washed many times with ethanol, dried, and used for another reaction with a similar method for 5 more runs. As a result, this catalyst can be recycled five times successfully with a slight decrease in activity (Fig. 3).

After the reaction, the IUST-1 catalyst was separated from the liquid and the supernatant was stirred for another 10 h at room temperature. GC/MS was used to analyze the products (see chromatogram graph as Fig. S8 in supplementary). Epoxide yield was preserved at 99.8% after the filtration stage, indicating no leakage of the cadmium catalyst. Moreover, a Cd metal leaching test was performed by ICP after the reaction, and the Cd concentration was below the detection limit, ruling out the possibility of Cd metal leaching. In addition, the structure of the recovered IUST-1 after the epoxidation of cyclooctene has been well maintained after five cycles, as shown by the PXRD patterns, FTIR spectra, and EDX analysis (see supplementary Figs. S5–S7 online).

Table 4 displays the comparison of the results of the performance of the IUST-1 with previously reported catalysts in the epoxidation of cyclooctene^34–42^. This prepared catalyst has advantages such as shorter reaction time, excellent catalytic activity, and high recycling capability.

**Table 4:** Comparison of the results acquired for epoxidation of cyclooctene catalyzed by the IUST-1 catalyst with some previous reports.

Entry	Catalyst	Support	Oxidant	Condition(°C)/solvent	Epoxide Conv%	Refs.
1	PW_12_/MIL-101	Cyclooctene	H_2_O_2_	Reflux/CH_3_CN	76	^34^
2	UiO-66-Mo(CO)_3_	Cyclooctene	TBHP	Reflux/CH_3_CN	98	^35^
3	Co-MOF-150-2	Cyclooctene	C_9_H_12_O_2_	Air/Reflux	78.5	^36^
4	PW-MOF^a^	Cyclooctene	H_2_O_2_	Reflux/CH_3_CN	83.5	^37^
5	Mo@UiO-67	Cyclooctene	TBHP	Reflux/ CH_2_Cl_2_	100	^38^
6	(THA)_2_[W_2_ O_3_(O_2_)_4_]	Cyclooctene	H_2_O_2_	Reflux/ CH_3_CN	100	^39^
7	TMU-16-NH_2_	Cyclooctene	TBHP	Reflux/CHCl_3_	83	^40^
8	UiO-66-NH_2_-SA-Mo	Cyclooctene	TBHP	Reflux/CH_2_CH_2_Cl_2_	97	^41^
9	CoPMA@UiO-bpy	Cyclooctene	H_2_O_2_	Reflux/CH_3_CN	91	^42^
10	**IUST-1**	**Cyclooctene**	**TBHP**	**Reflux/CCl**_**4**_	**99.8**	**This work**

^a^[Cu(Phen)(4,40-bpy) (H_2_O)]_2_[PW_12_O_40_] (4,40-bpy), (abbreviation: PW-MOF).

Significant values are given in Bold.

## Proposed mechanism for the epoxidation of olefin with TBHP by the IUST-1

A probable mechanism for the epoxidation of cyclooctene with TBHP has been proposed by the IUST-1 based on Sobczak’s ideas and other theoretical and experimental reports^43–46^. On the basis of this proposed mechanism, the first step in the epoxidation of an alkene is the coordination of the TBHP to the metal center by the terminal oxygen, thereby activating the peroxide for oxygen transfer (the observed trend for solvent effect also agrees with this mechanism. As the coordination ability of the solvent is increased, the solvent binding instead of TBHP binding to the metal center is also increased and the formation of species II is prevented and retarded the progress of the epoxidation reaction). The peroxidic oxygen in this species has an electrophilic character.

**Figure 4:**
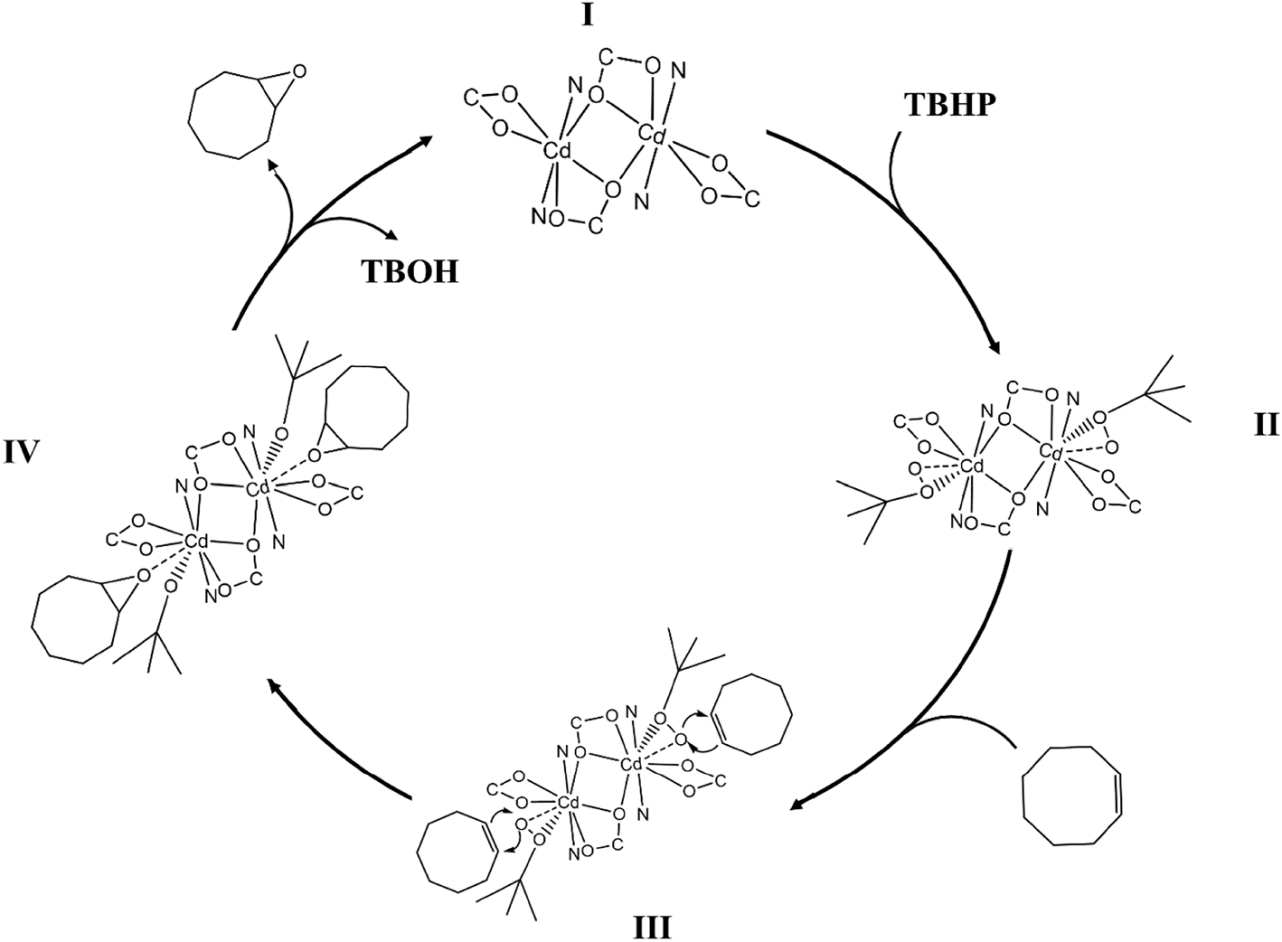
A schematic of the proposed mechanism for the reaction of cyclooctene oxidation in the presence of the IUST-1.

Then, the olefin substrate coordinates to the metal center and, as a nucleophile, inserts into the metal–oxygen bond of coordinated peroxide electrophile anion (the higher electron density of the double bond is expected to show more epoxidation reactivity). Then the epoxide was produced, and at the same time, the tert-butylperoxide anion converted to the tert-butoxide anion. After that, the peroxide product will be released and the cycle of catalytic reactions continues with the substitution of a new tert-butylperoxide instead of a tert-butoxide anion (Fig. 4).

## Conclusion

In conclusion, amino-functionalized Cd-MOF (IUST-1) based on thiazole ligand was prepared by reflux method as a fast, simple, and promising method for large scale and ultimately used for the epoxidation of olefins. The IUST-1 behaves as a very effective and selective heterogeneous catalysts for the epoxidation of olefins. The IUST-1 demonstrated a high yield (99.8%) using NH_4_VO_3_ as a co-catalyst. Meanwhile, FT-IR, EDX, and PXRD of the fresh and fifth recycled catalyst reuse demonstrated that the IUST-1 can efficiently catalyze the epoxidation of cyclooctene as a recyclable and stable heterogeneous catalyst. Moreover, in the presence of IUST-1, diverse types of olefins can be converted selectively into the corresponding epoxides with excellent selectivity and conversion.

### Supplementary Information

Supplementary Information.

## Data Availability

All data generated or analyzed during this study are included in this published article [and its supplementary information file].
