# Supplementary material for: Amino-induced cadmium metal–organic framework based on thiazole ligand as a heterogeneous catalyst for the epoxidation of alkenes
Source: Sci Rep. 2023 Sep 16;13:15391. doi: 10.1038/s41598-023-42666-1 (PMC10505202; doi:10.1038/s41598-023-42666-1)
Supplement: Supplementary file 1 — Supplementary Information. [file 41598_2023_42666_MOESM1_ESM.pdf]

Supplementary Information

Amino-induced cadmium metal-organic framework based on thiazole ligand as a heterogeneous catalyst for the epoxidation of alkenes

Fatemeh Moghadaskhou, Akram karballaee hossein, Azadeh Tadjarodi, *Mehdi Abroudi*

Research Laboratory of Inorganic Materials Synthesis, Department of Chemistry, Iran University of Science and Technology, 16846-13114, Tehran, Iran

** Corresponding author e-mail: tajarodi@iust.ac.ir (A. Tadjarodi)*

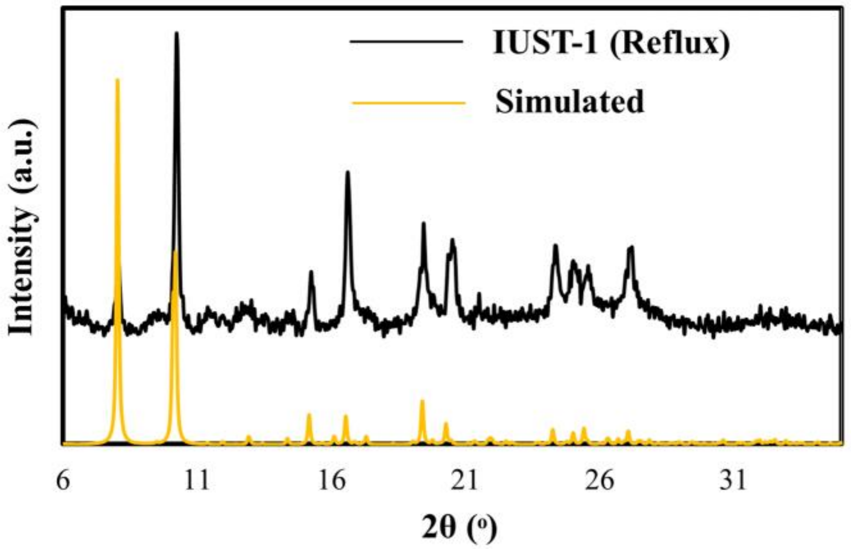

Figure S1. *PXRD patterns for IUST-1: Simulated, and prepared by the reflux process.*

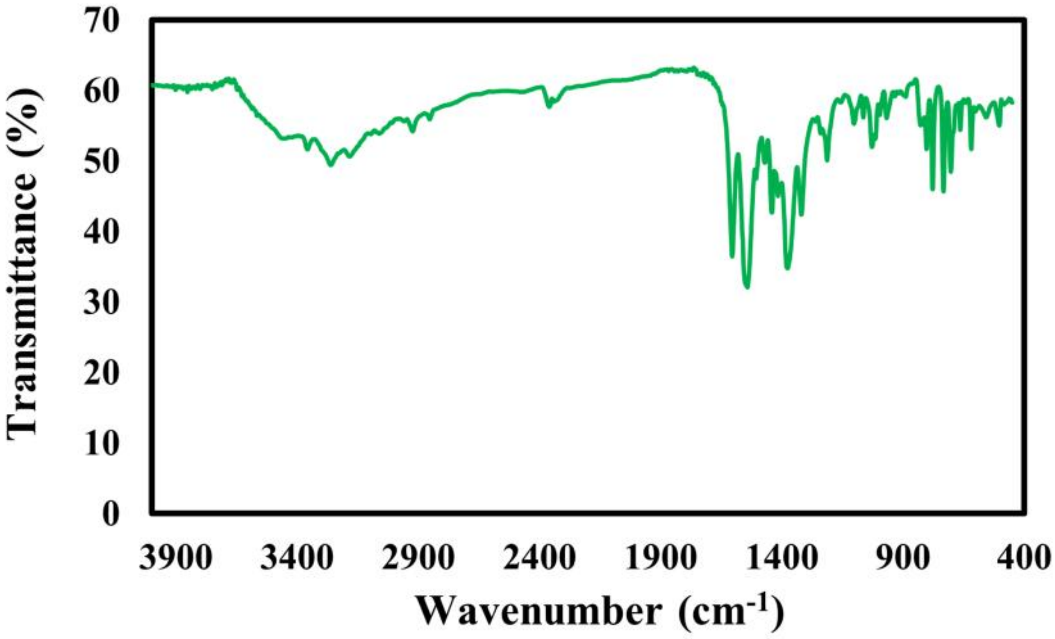

Figure S2. *FT-IR spectrum for IUST-1 prepared by the reflux process.*

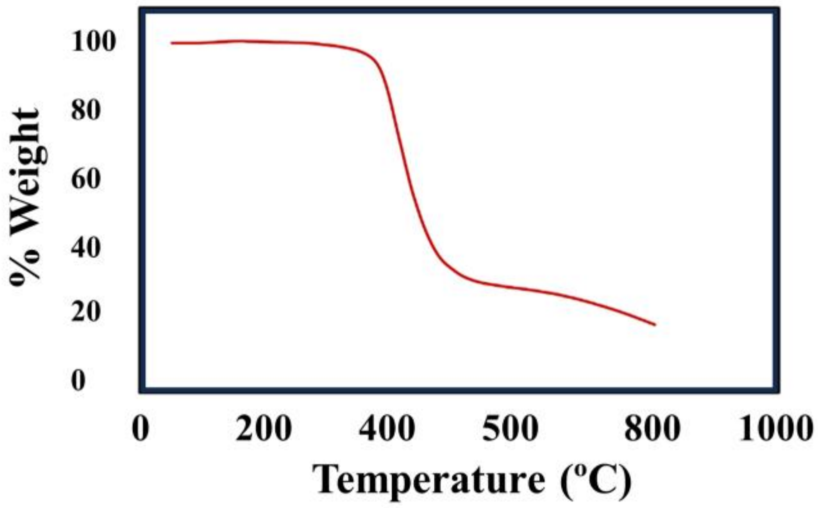

Figure S3. *TGA curve for IUST-1 prepared by the reflux process.*

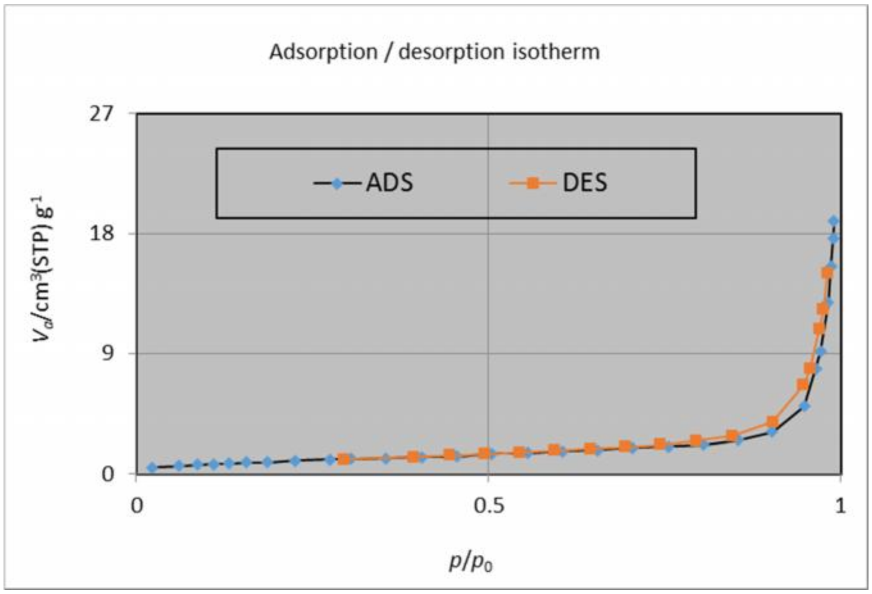

BET results	
V_m	*0.8031 [cm³(STP) g⁻¹]*
$a_{s,BET}$	*3.4955 [m² g⁻¹]*
C	*38.961*
Total pore volume(p/p₀=0.990)	*0.027906 [cm³ g⁻¹]*
Mean pore diameter	*31.933 [nm]*

Figure S4. *The adsorption-desorption isotherm and BET results for IUST-1.*

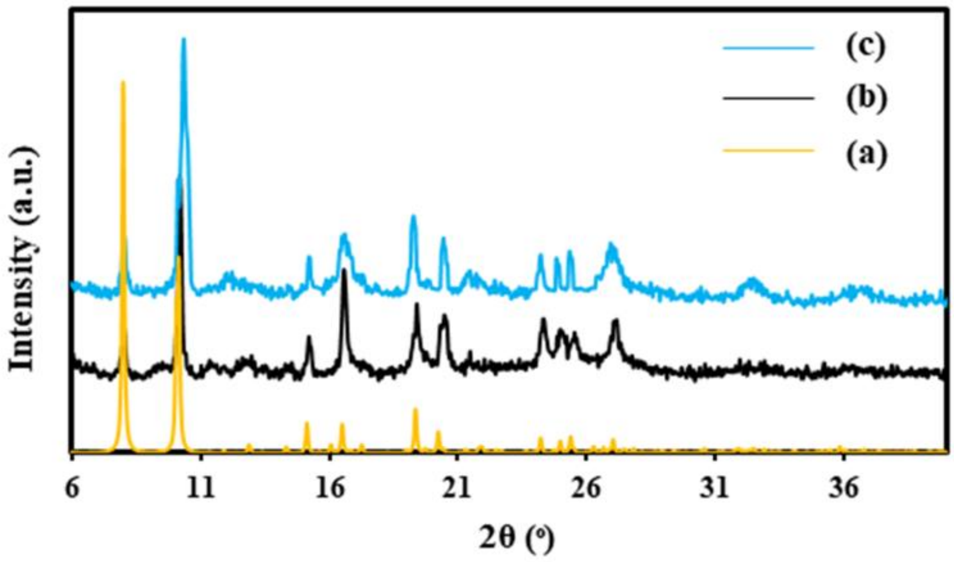

__Figure S5.__ PXRD patterns of IUST-1: a) Simulated b) prepared by the reflux process c) after 5 recycling.

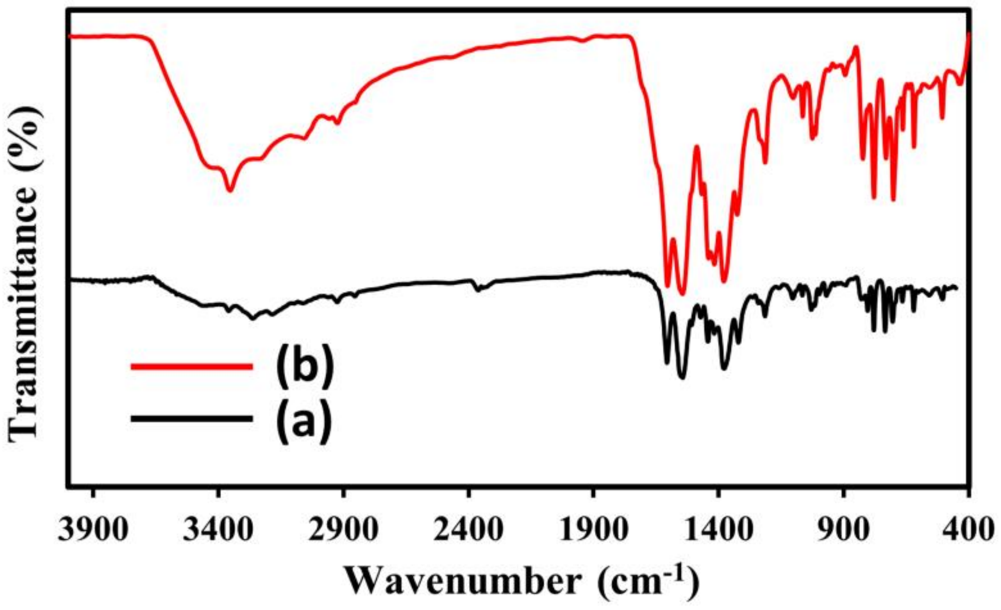

__Figure S6.__ FT-IR spectra of IUST-1: a) prepared by the reflux process b) after 5 recycling.

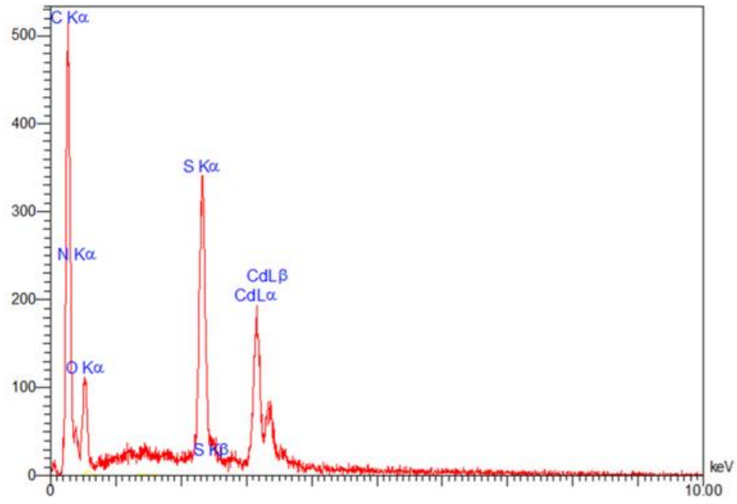

Element	Line	W%	A%
C	Ka	45.86	54.38
N	Ka	27.52	27.99
O	Ka	17.22	15.34
S	Ka	3.47	1.54
Cd	La	5.93	0.75
		100	100

Figure S7. *EDAX analysis for IUST-1 prepared by the reflux process after 5 recycling.*

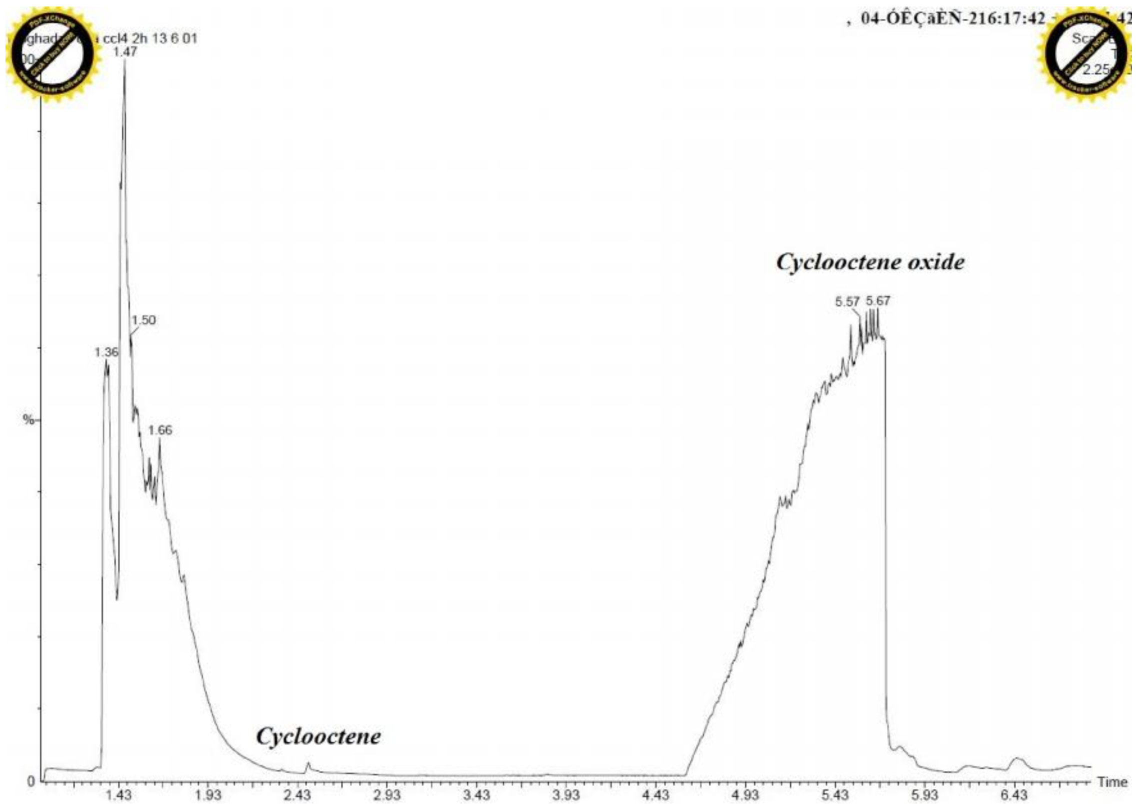

Figure S8. *Image taken from the GC Mass chromatogram graph for the catalytic reaction. (GC-MS samples were registered on a Shimadzu QP-5050 GC-MS device)*

Table S1. *Optimization of the value of the catalyst on the oxidation reaction[a] of cyclooctene.*

Entry	*IUST-1 (g)*	*Yield (%)*
**1**	0.005	48
**2**	0.01	73
**3**	0.015	89
**4**	**0.02**	**99.8**
**5**	0.025	99.8
**6**	0.03	99.8

[a]Reaction conditions: cyclooctene (1 mmol), CCl_4 (0.5 mL), NH_4VO_3 (4mg), TBHP (2 mmol), 76^oC, 2 hours.

Table S2. *Optimization of the time of the catalyst on the oxidation reaction[a] of cyclooctene.*

Entry	*Time (h)*	*Yield (%)*
**1**	0.5	40
**2**	1	73
**3**	**2**	**99.8**
**4**	3	99.8
**5**	4	99.8

[a]Reaction conditions: cyclooctene (1 mmol), 20mg IUST-1 as a catalyst, CCl_4 (0.5 mL), NH_4VO_3 (4mg), TBHP (2 mmol), 76^oC.

Table S3. *The role of NH_4VO_3 as co-catalysts on the oxidation reaction[a] of cyclooctene*

Entry	Catalysts	Yield (%)
**1**	NH_4VO_3	14
**2**	IUST-1	53
**3**	IUST-1+NH_4VO_3	99.8

[a]Reaction conditions: Substrate (1 mmol), 20mg IUST-1 as a catalyst, CCl_4 (0.5 mL), TBHP (2 mmol), 76°C.
